# Supplementary material for: Establishment of multi-stage intravenous self-administration paradigms in mice
Source: Sci Rep. 2022 Dec 11;12:21422. doi: 10.1038/s41598-022-24740-2 (PMC9742147; doi:10.1038/s41598-022-24740-2)
Supplement: Supplementary file 1 — Supplementary Information. [file 41598_2022_24740_MOESM1_ESM.docx]

**SUPPLEMENTARY MATERIAL**

**Figure S1. Cocaine self-administration training and testing paradigm.** Animals were trained to self-administer i.v. cocaine paired with a cue light by lever pressing in operant chambers. A schematic of the training and testing protocol is presented above. *Solid arrows* denote phases between which animals progressed automatically. *Dashed arrows* indicate that progression to the following phase was contingent on meeting predetermined behavioral criteria. Two levers were always available in the chambers. The ‘2 lever’ and 1 lever’ descriptors indicate the number of active, drug-delivering levers in the session. In 2lever sessions, both levers were active and responding on either resulted in a drug delivery. In 1lever sessions, one lever was active, and responding at this lever resulted in drug delivery, while the other lever was inactive. Responses at the inactive lever were recorded but had no programmed consequences. Advancement criteria were as follows: **(A)** ≥ 50 rewards; **(B)** ≥ 10 rewards in 2 consecutive sessions; **(C)** ≤ 9 rewards in 3 consecutive sessions; **(D)** Animals not meeting self-administration criteria after 4 overnight training sessions and twelve 1 hr, 2-lever, FR1 training sessions were terminated from study; **(E)** ≥ 10 rewards with ≥ 50% presses on the active lever in 2 consecutive sessions; **(F)** ≥ 2 sessions with ≥ 10 rewards, ≥ 50% presses on the active lever and ≤ 20% variability in rewards in the last 2 consecutive sessions OR ≥ 3 sessions with <10 rewards, ≥ 50% presses on the active lever and ≤ 20% variability in rewards in the last 2 consecutive sessions; **(G)** ≥ 3 sessions with ≥ 50% presses on the active lever and ≤ 20% variability in rewards in the last 2 consecutive sessions; **(H)** ≤ 20% of active lever presses in the last cocaine self-administration session.

**^€^**The lever selected as active was the non-preferred lever during last 2-lever training sessions.

Animals not meeting criteria for advancement repeated their current protocol until criteria were met. Failure to progress toward criteria after 4 consecutive sessions on the same protocol prompted a ketamine test for catheter patency.

**Figure S2. Remifentanil self-administration training and testing paradigm.** Animals were trained to self-administer i.v. remifentanil paired with a cue light by lever pressing in operant chambers. A schematic of the training and testing protocol is displayed above. *Solid arrows* denote phases between which animals progressed automatically. *Dashed arrows* indicate that progression to the following phase was contingent on meeting predetermined behavioral criteria. 2 levers were always available in the chambers. The ‘2 lever’ and ‘1 lever’ descriptors indicate the number of active, drug-delivering levers in the session. In 2 lever sessions, both levers were active and responding on either resulted in a drug delivery. In 1-lever sessions, one lever was active, and responding at this lever resulted in drug delivery, while the other lever was inactive. Responses at the inactive lever were recorded but had no programmed consequences. Advancement criteria were as follows: **(A)** ≥ 50 rewards; **(B)** ≥ 10 rewards in 2 consecutive sessions; **(C)** ≤ 9 rewards in 3 consecutive sessions; **(E)** Animals not meeting self-administration criteria after 4 overnight training sessions and twelve 1 hr, 2 lever, FR1 training sessions were excluded from study**; (E)** ≥ 10 rewards with ≥ 50% presses on the active lever in 2 consecutive sessions; **(F)** <10 rewards or <50% presses on the active lever in 6 consecutive sessions; **(G)** ≥ 10 rewards, ≥ 50% presses on the active lever in 2 consecutive sessions OR ≥ 3 sessions with <10 rewards, ≥ 50% presses on the active lever and ≤ 20% variability in rewards in the last 2 consecutive sessions; **(H)** ≥ 2 sessions with ≥ 10 rewards, ≥ 50% presses on the active lever and ≤ 20% variability in rewards in the last 2 consecutive sessions OR ≥ 3 sessions with <10 rewards, ≥ 50% presses on the active lever and ≤ 20% variability in rewards in the last 2 consecutive sessions; **(I)** ≥ 3 sessions with ≥ 50% presses on the active lever and ≤ 20% variability in rewards in the last 2 consecutive sessions.

**^€^**The lever selected as active was the non-preferred lever during last 2-lever training sessions.

Animals not meeting criteria for advancement repeated their current protocol until criteria were met. Failure to progress toward criteria after 4 consecutive sessions on the same protocol prompted a ketamine test for catheter patency.

**Figure S3. Exploratory and cocaine- and remifentanil-induced locomotor activities.** Exploratory and cocaine- or remifentanil-induced locomotor activities were evaluated in the open field using automated activity monitors. Mice were acclimated to the open field for 30 min prior to drug administration. Cocaine (20 mg/kg) or vehicle (saline) was injected (i.p.) and the mice were returned immediately to the open field for 60 min. After collecting baseline activity, the mice to receive remifentanil were injected (i.p.) sequentially with the vehicle (saline), followed at 20 min intervals by consecutive remifentanil treatments at doses of 0.1, 1.0 and 10.0 mg/kg (i.p.). In panels **(A)** and **(B)** group means ± SEM are represented by *green* (cocaine) or *purple* (remifentanil) *lines* and individual replicates by *gray lines*. Panels **(C-J)** are displayed as violin plots with individual data points represented by *grey circles*. Remifentanil data are shown in *purple* and cocaine data are depicted in *green*.

**(A)** Time-course of exploratory and cocaine-induced locomotion. Distance traveled (cm) in 5-min bins over the 60 min post-treatment period.

**(B)** Time-course of exploratory and remifentanil-induced locomotion. Distance traveled (cm) in 2-min bins over 20 min successive post-sequential treatments.

**(C)** Cumulative exploratory distance traveled.

**(D)** Mean exploratory velocity.

**(E)** Cumulative exploratory vertical episodes.

**(F)** Cumulative cocaine-induced distance traveled.

**(G)** Cumulative exploratory rest time.

**(H)** Cumulative exploratory center time.

**(I)** Cumulative exploratory stereotypic episodes.

**(J)** Cumulative remifentanil-induced distance traveled.

**Figure S4. Reinforcement dose-response curve by sex.** Data are represented as mean ± SEM.

**(A)** No main effect of sex on self-administered cocaine was identified. Data were analyzed using two-way, mixed model ANOVA. n = 20-28 males, 7-9 females. [F_Dose_(2.95, 88.39)=18.1, p<0.0001]; [F_Sex_(1, 36)=0.0004, p=0.9844]; [F_Interaction_(4, 120)=1.1, p=0.3588].

**(B)** No main effect of sex on self-administered remifentanil was observed. A significant dose x sex interaction, however, was identified. Data were analyzed using two-way, mixed model ANOVA. n = 12-26 males, 3-8 females. [F_Dose_(3.37, 85.03)=8.89, p<0.0001]; [F_Sex_(1, 32)=0.000929, p=0.9759]; [F_Interaction_(5, 126)=2.48, p= 0.0352].

n.s., not significant**.**

**Figure S5. Percent of total lever responses that occurred during the post-reinforcement time-out period by drug dose.** Data are represented as mean ± SEM. Percent time-out lever responses were fit to linear regressions (*solid lines*) with the 95% confidence limits of the best-fit line represented by *dashed lines*.

**(A)** In the cocaine paradigm, the percent of total lever responses that occurred during the time-out period decreased linearly with log cocaine dose. Y=-6.5X + 9.4. Slope 95% confidence interval: -9.0 to -4.1 time-out responses per log mg/kg/infusion cocaine.

**(B)** In the remifentanil paradigm, the percent of total lever responses that occurred during the time-out period was unrelated to the log remifentanil dose. Y=-.07X + 16.3. Slope 95% confidence interval: -2.2 to 0.9 time-out responses per log mg/kg/infusion remifentanil.

**Figure S6. Kinetics of self-administration over 60 min sessions.** Data are represented as mean ± SEM.

**(A)** Cumulative self-administered reinforcements for cocaine (0.1-3.0 mg/kg/infusion) over 60 min self-administration sessions.

**(B)** Cumulative drug intake for cocaine (0.1-3.0 mg/kg/infusion). Data for cocaine doses at and below 1.0 mg/kg/infusion were fit by linear regression. The 3.0 mg/kg/infusion cocaine dose curves were fit to rectangular hyperbolas.

**(C)** Cumulative self-administered reinforcements for remifentanil (0.01-3.0 mg/kg/infusion) over 60 min self-administration sessions.

**(D)** Cumulative drug intake for remifentanil (0.01-3.0 mg/kg/infusion).

Data for all remifentanil doses were fit by linear regression.

**Figure S7. Factor loading plot of variables in global factor analysis.** Loadings of 11 observed variables included in the global exploratory factor analysis on extracted factors 1 and 2 are presented. The term global is used to indicate that all animals were included in the analysis, regardless of the reinforcer/drug paradigm. Variable loading scores are included in **Table S4**.

**Figure S8. Machine learning regression model performance.** Absolute residual values from actual vs. predicted plots in **Figure 5** are presented as individual values (circles) and mean ± SEM (bars). Mean absolute error values between training and test sets were compared using *Student’s t*-*tests*.

**Table S1. Review of Studies on PubMed Using Cocaine or Opioid IVSA in Mice March 10, 2017-March 10, 2022^1^.**

| **#** | **PMID [Ref]** | **Drug Reinforcer** | **Strain(s)** | **Genotype^2^** | **Sex** | **Fixed ratios (FRs)** | **Doses**  **(mg/kg/**  **infusion)** | **Session length (min)** | **Extinction (Y/N)** | **Reinstate-ment (Y/N)** | **Prior Operant Food/Water Training (Y/N)** | **N/Group at Study Completion** | **Estimated Study Length (days)** | **Sex Difference Identified (Y/N)** |
| --- | --- | --- | --- | --- | --- | --- | --- | --- | --- | --- | --- | --- | --- | --- |
| **1** | 30771351 [17] | Cocaine | C57BL/6J | N/A | M | FR1 | 0, 0.05, 0.17, 0.5, 1.5, 4.5 | 120 | N | N | Y | 7 to 11 | Undeterminable | N/A |
| **2** | 33268545 [18] | Cocaine | C57BL/6J | Npas2 mutant  & WT | M & F | FR1 | 0.5 then 1.0, 0.5, 0.25, 0.125, 0.063, 0 (in a single session) | 60 | Y | Y | Y | 4 to 11 | 14 | Y (in mutant only) |
| **3** | 29423710 [14] | Cocaine | C57BL/6J | N/A | M | FR1 | 0.125, 0.25, 0.5, 1, 2 | 60 | Y | Y | N | 4 | 31 | N/A |
|  |  |  | C57BR/cdJ |  |  |  |  |  |  |  |  | 1 |  |  |
|  |  |  | I/LnJ |  |  |  |  |  |  |  |  | 8 |  |  |
|  |  |  | P/J |  |  |  |  |  |  |  |  | 4 |  |  |
|  |  |  | LG/J |  |  |  |  |  |  |  |  | 12 |  |  |
|  |  |  | FVB/NJ |  |  |  |  |  |  |  |  | 4 |  |  |
|  |  |  | LP/J |  |  |  |  |  |  |  |  | 10 |  |  |
|  |  |  | BTBR T+ tf/J |  |  |  |  |  |  |  |  | 4 |  |  |
| **4** | 28698920 [19] | Cocaine | Swiss Webster | N/A | M | FR1 | 1.0, 0.3 | 120 | N | N | N | 6 to 13 | Undeterminable | N/A |
| **5** | 33974940 [20] | Cocaine | C57BL/6J | N/A | M | FR1, FR3 | 0.25, 0.5 | 240 | N | N | N | 7 to 20 | 12 or 14 | N/A |
| **6** | 30798402 [21] | Cocaine | C57BL/6J | N/A | M | FR1 | 0.3 then 0.1, 0.03, .01, 0.003, 0.001 (in a single session) | 240 | N | N | N | 6 to 9 | 17 | N/A |
| **7** | 34133054 [22] | Cocaine | C57BL/6N | Fmr1 KO  & WT | M | FR1, FR3, FR5 | 0, 0.01, 0.03, 0.1, 0.32, 1.0, and 3.2 | 180 | Y | N | N | 5 to 6 | 17 to 43 | N/A |
| **8** | 34776891 [23] | Cocaine | C57BL/6J | N/A | M & F | FR1 | Cocaine: 0.75 | 120 | Y | Y | N | 10 to 11 | >26 | N |
|  |  | Heroin |  |  |  |  | Heroin: 0.1, 0.05, 0.025 |  |  |  |  | 9 to 22 |  |  |
| **9** | 31858986 [24] | Cocaine | C57BL/6 | A2a-Cre | M & F | FR1 | Cocaine | 120 | Y | Y | Y | 5 to 9 | 30 to 32 | Y |
| **10** | 31682894 [25] | Cocaine | C57BL/6N | Arc/Arg3.1 KO  & WT | M | FR1, FR2, FR3 | 0.01, 0.03, 0.1, 0.3, 1.0, 3.2 | 180 | Y | N | N | 6 to 7 | 18 to 58 | N/A |
| **11** | 32658535 [26] | Cocaine | C57BL/6J | N/A | M & F | FR2, FR3, FR5 | 1 | 120 | N | N | Y | 9 | 16 15 | N |
| **12** | 33839903 [27] | Cocaine | CD-1 | N/A | M | FR1 | 0.5 | 60 | Y | Y | N | 8 to 9 | Undeterminable | N/A |
| **13** | 32721403 [28] | Cocaine | C57BL/6J | FCG: XXF,XYF,  XXM,XYM | M & F | FR1 | 0.3, 0.6, 1.0 | 120 | N | N | N | 14 to 19 | 13 to 14 | Y |
| **14** | 34155331 [29] | Cocaine | C57BL/6J | N/A | M | FR1 | 0.5 | 60 | N | N | Y |  | 7 | N/A |
| **15** | 35229940 [30] | Cocaine | C57BL/6J | CalDAG-GEFI KO  & WT | M & F | FR1 | 0, 0.032, 0.1, 0.32, 1.0, 3.2 | 180 | Y | Y | Y | Not reported | Undeterminable | N |
| **16** | 28669705 [31] | Cocaine | C57BL/6J | N/A | M | FR1 | 0.5 | 120 | Y | Y | N | 29 |  |  |
| **17** | 31897574 [32] | Cocaine | CC004 & CC041 | N/A | M & F | FR1 | 0.56, 0.32, 0, 0.18, 0.1, 0.056, 0.032, 1.8 | 120 | Y | Y | N | 14 | <45 days | N |
| **18** | 32760242 [33] | Cocaine | C57BL6/J | N/A | M | FR1 | 0.5 | 120 | N | N | Y | 16 | 5 | N/A |
| **19** | 31162770 [34] | Cocaine | CD1 | N/A | M | FR1 | 0.75 | 120 | N | N | N | 10 | 12 | N/A |
| **20** | 32435819 [35] | Cocaine | C57BL6/J | N/A | M | FR1, FR3 | 0.5 | 120 | N | N | N | 6 to 11 | 9 to 14 | N/A |
| **21** | 30348770 [36] | Cocaine | Not reported | Heterozygous PTPRD-KO  & WT | Not reported | FR1 | 0.5 to 1.0 | 180 | N | N | N | Not reported | Undeterminable | N/A |
| **22** | 32049028 [37] | Cocaine | B6J.129S6(FVB) & B6129SF2/J | Vglut2-IRES-Cre, Penk-IRESCre & Vgat-IRES-Cre | M & F | FR1 | 0.75 | 120 | Y | Y | N | Not reported | >31 | N/A |
| **23** | 29250738 [38] | Cocaine | C57BL/6J | N/A | M | FR1 | 1 | 180 | Y | Y | N | Not reported | <20 | N/A |
| **24** | 32712273 [39] | Cocaine | Not reported | CB2 KO  & WT | M | FR1 | 0.5 | 180 | N | N | N | 19 | 8 to 12 | N/A |
| **25** | 30273593 [40] | Cocaine | CD1 | N/A | M | FR1 | 0.75 | 120 | Y | Y | N | 13 | 22 | N/A |
| **26** | 33046548 [41] | Opioid (Heroin) | C57BL/6J | DAT-IRES-Cre & vGAT-IRES-Cre | M & F | FR1 | 0.05, 0.025, 0.0125 | Not reported | Y | Y | N | 5 to 9 | Undeterminable | N |
| **27** | 33031806 [42] | Opioid (Oxycodone) | Not reported | A/A & G/G A112G MOR polymorphism | M & F | FR1 | 0.25 | 240 | N | N | N | 6 to 10 | 30 | Y |
| **28** | 30880124 [43] | Opioid (Heroin) | C57BL/6J | N/A | M & F | FR1 | 0.030 to 0.060 | 60 | N | N | N |  | <21 | Y |
| **29** | 32859725 [44] | Opioid (Remifentanil) | C57BL/6J | Cre-negative MOR floxed & D1-Cre, D2-Cre, A2a-Cre, or ChAT-Cre MOR floxed | M & F | FR1 | 0.05 | 120 | Y | N | N | 12 to 15 | <21 | N |
| **30** | 31058214 [45] | Opioid (Remifentanil) | Mixed (129, SJL backcrossed to C57BL/6J) | Nestin-Cre Glp1r floxed  & WT | M | FR1, FR3, FR5 | 0.01, 0.032 | 180 | Y | Y | N | 3 to 4 | Undeterminable | N/A |
| **31** | 32702404 [46] | Opioid (Oxycodone) | C57BL/6 | N/A | M & F | FR1 | 0.25 | 120 | N | N | N | 9 to 12 | 21 | Y |
| **32** | 31416242 [47] | Opioid (Remifentanil) | C57BL/6 | N/A | M | FR1 | 0.05 | 120 | Y | Y | Y | 14 | 30 | N/A |
|  |  | Opioid (Oxycodone) |  |  |  |  | 0.25 |  |  |  |  |  |  |  |

^1^On March 10, 2022, we entered the search terms ‘intravenous cocaine self-administration mice’ and ‘intravenous opioid self-administration mice’ into the PubMed database. Results were limited to the last 5 years from the search date. All articles returned by this search were systematically evaluated by a team of 4 investigators. Articles returned by this search not containing cocaine or opioid IVSA in mice were excluded from the table. N/Group at Study Completion and estimates of Study Length were based on available information in Methods, Results, Figures, Figure Legends, and/or Supplemental Information. For some articles, insufficient information was provided to make an estimate. These cases are denoted by ‘Not reported’ or ‘Undeterminable’.

^2^KO, knockout; WT, wild-type; N/A, not applicable

**Table S2. Self-administration acquisition – Supporting Figure 2.**

|  | Cocaine | Remifentanil | P value* |
| --- | --- | --- | --- |
| Sessions to meet extended access criteria | 1.2 ± 0.4 | 1.1 ± 0.4 | 0.0931 |
| Sessions to meet 2 lever, FR1 criteria | 3.3 ± 2.0 | 2.2 ± 0.4 | 0.0010 |
| Sessions to meet 1 lever, FR1 criteria | 4.0 ± 1.6 | 2.9 ± 0.8 | 0.0009 |
| Sessions to meet 1 lever, FR2 criteria | 2.1 ± 0.5 | 2.0 ± 0.2 | 0.4767 |
| Sessions to meet 1 lever, FR4 criteria | 2.8 ± 1.6 | 2.6 ± 0.8 | 0.7891 |
| Total required training sessions | 13.5 ± 3.8 | 10.8 ± 1.2 | 0.0002 |
| Mice trained (n) | 38 | 34 |  |
| Mice failed to train (n) | 7 | 2 |  |
| Percent mice completing training | 84.4 | 94.4 | 0.1547^¥^ |

Mean number of sessions required to meet specific criteria ± SD.

*Mann-Whitney test.

¥Chi-square test.

**Table S3. Statistical analyses of data presented in Figure 2 – Supporting Figure 2.**

| **Figure** | **Panel** | **Experiment Description** | **F Statistics^1^** | **Multiple Comparisons^2^** | **N^3^** | **Curve Fit** | **Parameter** | **95% CI** | **Units** |
| --- | --- | --- | --- | --- | --- | --- | --- | --- | --- |
| **2** | **A** | Number and proportion of mice that met final SA acquisition criteria,  **Cocaine** |  |  | 42 |  |  |  |  |
|  | **B** | Number of sessions required to meet training criteria, **Cocaine** |  |  | 38 | Nonlinear regression, Lorentizian fit | Center  Width  R^2^  Mean  Median  SD | 8.1 to 12.7  0.9 to 7.7  0.6093  13.5  12.0  3.8 | sessions  sessions  sessions  sessions |
|  | **C** | Number and proportion of mice that met final SA acquisition criteria,  **Remifentanil** |  |  | 36 |  |  |  |  |
|  | **D** | Number of sessions required to meet training criteria,  **Remifentanil** |  |  | 34 | Nonlinear regression, Lorentizian fit | Center  Width  R^2^  Mean  Median  SD | 10.0 to 11.1  -2.3 to 2.3  0.9060  10.8  11  1.3 | sessions  sessions  sessions  sessions |
|  | **E** | Lever responses vs. training session type, **Cocaine** | F_Session_(3.34, 200.2)=23.7, p<0.0001  F_Lever_(1,74)=32.4, p<0.0001  F_Interaction_(8, 480)=49.7, p<0.0001  Geisser-Greenhouse’s ε=0.42 | ***p<0.0001, Active vs. Inactive Lever | 38 |  |  |  |  |
|  | **F** | Lever responses vs. training session type, **Remifentanil** | F_Session_(4.35, 208.4)=15.1, p<0.0001  F_Lever_(1,66)=44.3, p<0.0001  F_Interaction_(8, 383)=33.9, p<0.0001  Geisser-Greenhouse’s ε=0.54 | ***p<0.0001, Active vs. Inactive Lever | 34 |  |  |  |  |
|  | **G** | Active lever responses vs. FR,  **Cocaine** | F(1.55, 55.87)=71.0, p<0.0001  Geisser-Greenhouse’s ε=0.89 | ***p<0.001, FR1 vs. FR2, FR1 vs. FR4, FR2 vs. FR4 | 38 |  |  |  |  |
|  | **H** | Inactive lever responses vs. FR, **Cocaine** | F(1.80, 64.51)=6.234, p=0.0045  Geisser-Greenhouse’s ε=0.90 | *p<0.05, FR1 vs. FR2  *p<0.01, FR1 vs. FR4 | 38 |  |  |  |  |
|  | **I** | Active lever responses vs. FR,  **Remifentanil** | F(1.80, 57.65)=33.3, p<0.0001  Geisser-Greenhouse’s ε=0.90 | *p<0.01, FR1 vs. FR2  ***p<0.0001, FR1 vs. FR4, FR2 vs. FR4 | 34 |  |  |  |  |
|  | **J** | Inactive lever responses vs. FR, **Remifentanil** | F(1.945, 62.23)=0.7, p=0.5019  Geisser-Greenhouse’s ε=0.97 | n.s. | 34 |  |  |  |  |
|  | **K** | Lever accuracy vs. FR, **Cocaine** | F(1.95, 70.20)=5.4, p=0.0072  Geisser-Greenhouse’s ε=0.98 | *p<0.01, FR1 vs. FR4 | 38 |  |  |  |  |
|  | **L** | Reinforcements vs. FR, **Cocaine** | F(1.77, 63.77)=20.5, p<0.0001  Geisser-Greenhouse’s ε=0.89 | *p<0.05, FR1 vs. FR2  ***p<0.001, FR1 vs. FR4  **p<0.001 FR2 vs. FR4 | 38 |  |  |  |  |
|  | **M** | Lever accuracy vs. FR, **Remifentanil** | F(1.97, 63.09)=5.6, p=0.0057  Geisser-Greenhouse’s ε=0.96 | *p<0.01 FR1 vs. FR4 | 34 |  |  |  |  |
|  | **N** | Reinforcements vs. FR, **Remifentanil** | F(1.94, 62.03)=35.5, p<0.0001  Geisser-Greenhouse’s ε=0.97 | *p<0.05, FR1 vs. FR2  ***p<0.0001, FR1 vs. FR4, FR2 vs. FR4 | 34 |  |  |  |  |

^1^F statistics from two-way, mixed effects analyses for panels E and F and one-way, repeated measures ANOVAs for panels G-L.

^2^*Post-hoc* Sidak tests for panels E and F and Tukey’s multiple comparisons tests for panels G-L.

^3^A single mouse in each of the cocaine and remifentanil paradigms was removed from training analyses because they were prematurely progressed to FR4 due to experimenter error. Sphericity was not assumed, and the analyses were corrected using the Geisser-Greenhouse epsilon (ε) hat method.

Abbreviations: CI, confidence internal; SD, standard deviation; n.s., not significant.

**Table S4. Curve parameters presented in Figure 3 – Supporting Figure 3.**

| **Figure** | **Panel** | **Experiment Description** | **Curve Fit** | **N** | **Parameter** | **95% CI** | **Units** |
| --- | --- | --- | --- | --- | --- | --- | --- |
| **3** | **A** | Earned reinforcements vs. log cocaine dose | Nonlinear regression, second order polynomial | 27-38 | B0  B1  B2  R^2^ | 13.8 to 16.5  -13.3 to -7.1  13.6 to -5.4  0.2042 | mg/kg/infusion cocaine  mg/kg/infusion cocaine  mg/kg/infusion cocaine |
|  | **B** | Total active lever responses vs. log cocaine dose | Nonlinear regression, second order polynomial | 27-38 | B0  B1  B2  R^2^ | 58.8 to 68.7  56.6 to -33.0  -40.6 to -10.1  0.1481 | mg/kg/infusion cocaine  mg/kg/infusion cocaine  mg/kg/infusion cocaine |
|  | **C** | Total inactive lever responses vs. log cocaine dose | Linear regression | 27-38 | Slope  Y-intercept  R^2^ | 15.9 to -10.1  9.34 to 12.66  0.1644 | lever response per mg/kg/infusion cocaine  lever responses |
|  | **D** | Cocaine consumed vs. log cocaine dose | Nonlinear regression, sigmoidal | 27-38 | Bottom  Top  EC_50_  Hill Slope  R^2^ | Constrained to 0  30.6 to 61.8  1.0 to 4.7  0.8 to 1.3  0.7704 | mg/kg cocaine  mg/kg/infusion cocaine |
|  | **E** | Percent active lever responses vs. log cocaine dose | Linear regression | 27-38 | Slope  Y-intercept  R^2^ | 7.5 to 14.4  84.9 to 88.8  0.1942 | percent active lever responses per mg/kg/infusion cocaine  percent active lever responses |
|  | **F** | Latency to first lever response vs. log cocaine dose | Linear regression | 27-38 | Slope  Y-intercept  R^2^ | -32.9 to 14.89  27.9 to 55.2  0.003387 | sec per mg/kg/infusion cocaine  sec |
|  | **G** | Latency to first earned reinforcement vs. log cocaine dose | Linear regression | 27-38 | Slope  Y-intercept  R^2^ | 174.1 to -54.98  149.9 to 217.9  0.08126 | sec per mg/kg/infusion cocaine  sec |
|  | **H** | Post-reinforcement time-out responses vs. log cocaine dose | Linear regression | 27-38 | Slope  Y-intercept  R^2^ | -17.6 to -7.4  5.2 to 11.0  0.1252 | time-out responses per mg/kg/infusion cocaine  time-out responses |
|  | **I** | Earned reinforcements vs. log remifentanil dose | Nonlinear regression, second order polynomial | 15-34 | B0  B1  B2  R^2^ | 17.34 to 21.38  -17.03 to -8.53  -7.71 to 3.05  0.1945 | mg/kg/infusion remifentanil  mg/kg/infusion remifentanil  mg/kg/infusion remifentanil |
|  | **J** | Total active lever responses vs. log remifentanil dose | Nonlinear regression, second order polynomial | 15-34 | B0  B1  B2  R^2^ | 82.77 to 106.8  -88.45 to -37.86  -37.22 to -9.45  0.1671 | mg/kg/infusion remifentanil  mg/kg/infusion remifentanil  mg/kg/infusion remifentanil |
|  | **K** | Total inactive lever responses vs. log remifentanil dose | Linear regression | 15-34 | Slope  Y-intercept  R^2^ | -13.15 to -6.58  7.41 to 15.24  0.1733 | lever response per mg/kg/infusion remifentanil  lever responses |
|  | **L** | Remifentanil consumed vs. log remifentanil dose | Nonlinear regression, exponential growth | 15-34 | Y0  k  Tau  Doubling Time  R^2^ | 16.26 to 18.63  1.68 to 2.00  0.50 to 0.59  0.35 to 0.41  0.8791 | (mg/kg/infusion remifentanil)^-1^  mg/kg/infusion remifentanil |
|  | **M** | Percent active lever responses vs. log remifentanil dose | Linear regression | 15-34 | Slope  Y-intercept  R^2^ | 3.21 to 6.84  88.30 to 92.63  0.1506 | percent active lever responses per mg/kg/infusion remifentanil  percent active lever responses |
|  | **N** | Latency to first lever response vs. log remifentanil dose | Linear regression | 15-34 | Slope  Y-intercept  R^2^ | -5.18 to 4.63  6.27 to 18.00  7.334e-005 | sec per mg/kg/infusion  sec |
|  | **O** | Latency to first earned reinforcement vs. log remifentanil dose | Linear regression | 15-34 | Slope  Y-intercept  R^2^ | -48.00 to -15.53  16.79 to 55.41  0.08246 | sec per mg/kg/infusion  sec |
|  | **P** | Post-reinforcement time-out responses vs. log remifentanil dose | Linear regression | 15-34 | Slope  Y-intercept  R^2^ | -14.50 to -5.29  12.86 to 23.85  0.09676 | responses per mg/kg/infusion  cue responses |

Abbreviations: CI, confidence interval; sec, seconds.

**Table S5. Curve parameters and statistical analyses of data presented in Figure 4 – Supporting Figure 4.**

| **Figure** | **Panel** | **Experiment Description** | **F/t Statistics^1^** | **Multiple Comparisons^2^** | **N** | **Curve Fit** | **Parameter** | **95% CI** | **Units** |
| --- | --- | --- | --- | --- | --- | --- | --- | --- | --- |
| **4** | **A** | Active and inactive lever responses vs. extinction session number,  **Cocaine** | F_Session_(3.16, 156.6)=34.4, p<0.0001  F_Lever_(1,64)=38.6, p<0.0001  F_Interaction_(21, 1042)=15.5, p<0.0001  Geisser-Greenhouse’s ε=0.15 | *p<0.001-p<0.05 Active vs. Inactive Lever | 14-32 |  |  |  |  |
|  | **B** | Total lever presses vs. extinction session number,  **Cocaine** |  |  | 14-32 | Nonlinear regression, exponential one-phase decay | Y0  Plateau  K  Half Life  Tau  R^2^ | 202.1 to 296.7  36.19 to 43.80  0.4977 to 0.8295  0.8356 to 1.393  1.206 to 2.009  0.4218 | lever responses  lever responses  (session)^-1^  session  session |
|  | **C, left** | Lever discrimination: Active SA vs first extinction session,  **Cocaine** | W = -458, p***<0.0001 |  | 31 |  |  |  |  |
|  | **C, middle** | Lever discrimination: First vs. Last extinction session,  **Cocaine** | W = -367, p***<0.0001 |  | 29 |  |  |  |  |
|  | **C, right** | Lever discrimination: Last extinction session vs. Reinstatement session,  **Cocaine** | W = 339, p***<0.0001 |  | 29 |  |  |  |  |
|  | **D** | Active and inactive lever responses by session type,  **Cocaine** | F_Session_(1.86,109.8)=76.0, p<0.0001  F_Lever_(1,64)=98.5, p<0.0001  F_Interaction_(2,118)=93.9, p<0.0001  Geisser-Greenhouse’s ε=0.93 | ***p<0.0001, Active Lever SA vs. Active Lever Extinction; Active Lever Extinction vs. Active Lever Reinstatement  ###p<0.0001 Active vs. Inactive Lever | 29-32 |  |  |  |  |
|  | **E** | Active and inactive lever responses vs. extinction session number, **Remifentanil** | F_Session_(3.78, 176.7)=60.9, p<0.0001  F_Lever_(1,56)=37.2, p<0.0001  F_Interaction_(20, 936)=30.5, p<0.0001  Geisser-Greenhouse’s ε=0.19 | *p<0.001-p<0.05 Active vs. Inactive Lever | 22-28 |  |  |  |  |
|  | **F** | Total lever presses vs. extinction session number,  **Remifentanil** |  |  | 22-28 | Nonlinear regression, exponential one-phase decay | Y0  Plateau  K  Half Life  Tau  R^2^ | 300.2 to 400.8  36.63 to 48.19  0.4086 to 0.6137  1.129 to 1.696  1.630 to 2.447  0.4967 | lever responses  lever responses  (session)^-1^  session  session |
|  | **G, left** | Lever discrimination: Active SA vs first extinction session,  **Remifentanil** | W = -340, p***<0.0001 |  | 28 |  |  |  |  |
|  | **G, middle** | Lever discrimination: First vs. Last extinction session,  **Remifentanil** | W = -153, p*<0.0115 |  | 22 |  |  |  |  |
|  | **G, right** | Lever discrimination: Last extinction session vs. Reinstatement session,  **Remifentanil** | W = 139, p*<0.0224 |  | 22 |  |  |  |  |
|  | **H** | Active and inactive lever responses by session type,  **Remifentanil** | F_Session_(1.98,85.15)=20.4, p<0.0001  F_Lever_(1,58)=67.2, p<0.0001  F_Interaction_(2,868)=21.7, p<0.0001  Geisser-Greenhouse’s ε=0.99 | ***p<0.0001, Active Lever SA vs. Active Lever Extinction; Active Lever Extinction vs. Active Lever Reinstatement  ###p<0.0001 Active vs. Inactive Lever | 22-29 |  |  |  |  |

^1^F statistics from two-way, mixed effects analyses and W statistics from two-tailed, Wilcoxen Matched-Pairs Test.

^2^*Post-hoc* Sidak tests for panels A and E, and Tukey’s multiple comparisons tests for panels D and H. Sphericity was not assumed and the analyses were corrected using the Geisser-Greenhouse epsilon (ε) hat method.

Abbreviations: CI, confidence internal; SD, standard deviation; n.s., not significant.

**Table S6. Correlation matrix for cocaine taking and cocaine seeking assessments – Supporting Figure 5.**

Significance level (one-tailed)

Pearson Correlation Coefficients

|  |  | Acquisition Sessions Required | Reinforce-ments | Active Lever Responding | Inactive Lever Responding | Time-out Responding | Accuracy | Latency First Response | Latency First Reward | Early Extinction | Late Extinction | Reinstate-ment | Extinction Sessions Required |
| --- | --- | --- | --- | --- | --- | --- | --- | --- | --- | --- | --- | --- | --- |
|  | Acquisition Sessions Required | 1 | -0.037 | -0.073 | 0.242 | 0.218 | -0.237 | 0.337 | 0.427 | -0.135 | 0.223 | -0.211 | 0.316 |
|  | Reinforcements | -0.037 | 1 | 0.915 | 0.23 | 0.359 | 0.312 | -0.38 | -0.579 | 0.636 | 0.101 | 0.32 | -0.127 |
|  | Active Lever Responding | -0.073 | 0.915 | 1 | 0.143 | 0.514 | 0.373 | -0.41 | -0.59 | 0.619 | 0.038 | 0.316 | -0.181 |
|  | Inactive Lever Responding | 0.242 | 0.23 | 0.143 | 1 | 0.322 | -0.768 | -0.223 | -0.016 | 0.11 | 0.245 | 0.055 | 0.232 |
|  | Time-out Responding | 0.218 | 0.359 | 0.514 | 0.322 | 1 | -0.099 | -0.079 | -0.054 | 0.145 | 0.16 | 0.084 | 0.19 |
|  | Accuracy | -0.237 | 0.312 | 0.373 | -0.768 | -0.099 | 1 | 0.01 | -0.291 | 0.223 | -0.182 | 0.028 | -0.298 |
|  | Latency first response | 0.337 | -0.38 | -0.41 | -0.223 | -0.079 | 0.01 | 1 | 0.793 | -0.244 | -0.129 | -0.013 | -0.046 |
|  | Latency first reward | 0.427 | -0.579 | -0.59 | -0.016 | -0.054 | -0.291 | 0.793 | 1 | -0.497 | -0.067 | -0.018 | 0.113 |
|  | Early Extinction | -0.135 | 0.636 | 0.619 | 0.11 | 0.145 | 0.223 | -0.244 | -0.497 | 1 | -0.033 | 0.249 | -0.183 |
|  | Late Extinction | 0.223 | 0.101 | 0.038 | 0.245 | 0.16 | -0.182 | -0.129 | -0.067 | -0.033 | 1 | -0.101 | 0.741 |
|  | Reinstatement | -0.211 | 0.32 | 0.316 | 0.055 | 0.084 | 0.028 | -0.013 | -0.018 | 0.249 | -0.101 | 1 | -0.107 |
|  | Extinction Sessions Required | 0.316 | -0.127 | -0.181 | 0.232 | 0.19 | -0.298 | -0.046 | 0.113 | -0.183 | 0.741 | -0.107 | 1 |
|  |  |  |  |  |  |  |  |  |  |  |  |  |  |
|  | Acquisition Sessions Required |  | 0.414 | 0.333 | 0.075 | 0.098 | 0.079 | 0.021 | 0.004 | 0.212 | 0.093 | 0.105 | 0.029 |
|  | Reinforcements | 0.414 |  | 0 | 0.082 | 0.013 | 0.028 | 0.009 | 0 | 0 | 0.274 | 0.025 | 0.224 |
|  | Active Lever Responding | 0.333 | 0 |  | 0.195 | 0 | 0.011 | 0.005 | 0 | 0 | 0.411 | 0.027 | 0.138 |
|  | Inactive Lever Responding | 0.075 | 0.082 | 0.195 |  | 0.025 | 0 | 0.089 | 0.461 | 0.255 | 0.069 | 0.371 | 0.081 |
|  | Time-out Responding | 0.098 | 0.013 | 0 | 0.025 |  | 0.277 | 0.319 | 0.373 | 0.192 | 0.169 | 0.309 | 0.126 |
|  | Accuracy | 0.079 | 0.028 | 0.011 | 0 | 0.277 |  | 0.475 | 0.038 | 0.089 | 0.136 | 0.433 | 0.034 |
|  | Latency first response | 0.021 | 0.009 | 0.005 | 0.089 | 0.319 | 0.475 |  | 0 | 0.07 | 0.22 | 0.47 | 0.393 |
|  | Latency first reward | 0.004 | 0 | 0 | 0.461 | 0.373 | 0.038 | 0 |  | 0.001 | 0.345 | 0.457 | 0.249 |
|  | Early Extinction | 0.212 | 0 | 0 | 0.255 | 0.192 | 0.089 | 0.07 | 0.001 |  | 0.429 | 0.085 | 0.158 |
|  | Late Extinction | 0.093 | 0.274 | 0.411 | 0.069 | 0.169 | 0.136 | 0.22 | 0.345 | 0.429 |  | 0.291 | 0 |
|  | Reinstatement | 0.105 | 0.025 | 0.027 | 0.371 | 0.309 | 0.433 | 0.47 | 0.457 | 0.085 | 0.291 |  | 0.284 |
|  | Extinction Sessions Required | 0.029 | 0.224 | 0.138 | 0.081 | 0.126 | 0.034 | 0.393 | 0.249 | 0.158 | 0 | 0.284 |  |

**Table S7. Correlation matrix for remifentanil taking and remifentanil seeking assessments – Supporting Figure 5.**

Pearson Correlation Coefficients

Significance level (one-tailed)

|  |  | Acquisition Sessions Required | Reinforce-ments | Active Lever Responding | Inactive Lever Responding | Time-out Responding | Accuracy | Latency First Response | Latency First Reward | Early Extinction | Late Extinction | Reinstate-ment |
| --- | --- | --- | --- | --- | --- | --- | --- | --- | --- | --- | --- | --- |
|  | Acquisition Sessions Required | 1 | 0.261 | 0.18 | -0.201 | -0.049 | 0.272 | 0.019 | -0.049 | -0.306 | -0.388 | -0.349 |
|  | Reinforcements | 0.261 | 1 | 0.94 | 0.327 | 0.13 | 0.198 | -0.474 | -0.485 | 0.178 | 0.111 | -0.045 |
|  | Active Lever Responding | 0.18 | 0.94 | 1 | 0.395 | 0.422 | 0.163 | -0.396 | -0.425 | 0.318 | 0.156 | -0.037 |
|  | Inactive Lever Responding | -0.201 | 0.327 | 0.395 | 1 | 0.07 | -0.783 | -0.186 | -0.138 | 0.387 | 0.395 | 0.273 |
|  | Time-out Responding | -0.049 | 0.13 | 0.422 | 0.07 | 1 | 0.15 | 0.101 | -0.005 | 0.268 | 0.088 | -0.036 |
|  | Accuracy | 0.272 | 0.198 | 0.163 | -0.783 | 0.15 | 1 | 0.005 | -0.105 | -0.238 | -0.365 | -0.287 |
|  | Latency first response | 0.019 | -0.474 | -0.396 | -0.186 | 0.101 | 0.005 | 1 | 0.853 | -0.188 | -0.143 | -0.114 |
|  | Latency first reward | -0.049 | -0.485 | -0.425 | -0.138 | -0.005 | -0.105 | 0.853 | 1 | -0.153 | -0.064 | -0.124 |
|  | Early Extinction | -0.306 | 0.178 | 0.318 | 0.387 | 0.268 | -0.238 | -0.188 | -0.153 | 1 | 0.722 | 0.36 |
|  | Late Extinction | -0.388 | 0.111 | 0.156 | 0.395 | 0.088 | -0.365 | -0.143 | -0.064 | 0.722 | 1 | 0.601 |
|  | Reinstatement | -0.349 | -0.045 | -0.037 | 0.273 | -0.036 | -0.287 | -0.114 | -0.124 | 0.36 | 0.601 | 1 |
|  |  |  |  |  |  |  |  |  |  |  |  |  |
|  | Acquisition Sessions Required |  | 0.071 | 0.158 | 0.131 | 0.394 | 0.063 | 0.458 | 0.394 | 0.042 | 0.013 | 0.023 |
|  | Reinforcements | 0.071 |  | 0 | 0.03 | 0.233 | 0.131 | 0.002 | 0.002 | 0.156 | 0.266 | 0.399 |
|  | Active Lever Responding | 0.158 | 0 |  | 0.01 | 0.006 | 0.178 | 0.01 | 0.006 | 0.033 | 0.189 | 0.418 |
|  | Inactive Lever Responding | 0.131 | 0.03 | 0.01 |  | 0.348 | 0 | 0.146 | 0.218 | 0.012 | 0.01 | 0.059 |
|  | Time-out Responding | 0.394 | 0.233 | 0.006 | 0.348 |  | 0.198 | 0.285 | 0.49 | 0.063 | 0.309 | 0.419 |
|  | Accuracy | 0.063 | 0.131 | 0.178 | 0 | 0.198 |  | 0.49 | 0.278 | 0.088 | 0.017 | 0.05 |
|  | Latency first response | 0.458 | 0.002 | 0.01 | 0.146 | 0.285 | 0.49 |  | 0 | 0.144 | 0.209 | 0.26 |
|  | Latency first reward | 0.394 | 0.002 | 0.006 | 0.218 | 0.49 | 0.278 | 0 |  | 0.193 | 0.359 | 0.242 |
|  | Early Extinction | 0.042 | 0.156 | 0.033 | 0.012 | 0.063 | 0.088 | 0.144 | 0.193 |  | 0 | 0.028 |
|  | Late Extinction | 0.013 | 0.266 | 0.189 | 0.01 | 0.309 | 0.017 | 0.209 | 0.359 | 0 |  | 0.001 |
|  | Reinstatement | 0.023 | 0.399 | 0.418 | 0.059 | 0.419 | 0.05 | 0.26 | 0.242 | 0.028 | 0.001 |  |

**Table S8. Factor loadings of variables from exploratory factor analyses – Supporting Figure 6.**

|  | **Within-Drug Cocaine** | | | **Within-Drug Remifentanil** | | **Global** | |  |
| --- | --- | --- | --- | --- | --- | --- | --- | --- |
| **Variable** | **Factor 1** | **Factor 2** | | **Factor 1** | **Factor 2** | **Factor 1** | **Factor 2** | |
| Acquisition sessions required | -0.072 | 0.270 | 0.179 | | -0.296 | -0.326 | 0.147 | |
| Reinforcements* | 0.952 | 0.011 | 0.941 | | -0.054 | 0.925 | 0.075 | |
| Active lever responding* | 0.964 | -0.073 | 0.999 | | -0.004 | 0.991 | 0.060 | |
| Inactive lever responding* | 0.224 | 0.945 | 0.399 | | 0.889 | 0.321 | 0.885 | |
| Percent time-out responses* | 0.454 | 0.249 | 0.419 | | -0.091 | 0.551 | 0.121 | |
| Lever accuracy* | 0.323 | -0.889 | 0.159 | | -0.951 | 0.522 | -0.818 | |
| Latency to first lever response* | -0.433 | -0.136 | -0.397 | | -0.052 | -0.417 | -0.145 | |
| Latency to first reinforcement* | -0.613 | 0.122 | -0.426 | | 0.033 | -0.660 | 0.068 | |
| Early extinction lever responding | 0.649 | -0.035 | 0.319 | | 0.314 | 0.527 | 0.204 | |
| Late extinction lever responding | 0.072 | 0.253 | 0.158 | | 0.408 | 0.229 | 0.344 | |
| Reinstatement lever responding | 0.311 | 0.010 | -0.035 | | 0.325 | 0.233 | 0.191 | |
| Extinction sessions required | -0.150 | 0.296 | N/A | | N/A | N/A | N/A | |
| **Eigenvalue**^1^ | 3.728 | 2.562 | 3.396 | | 2.673 | 4.195 | 2.004 | |
| **% Variance**^1^ | 31.069 | 21.351 | 30.871 | | 24.300 | 38.140 | 18.219 | |
| **Cumulative %**^1^ | 52.420 | | 55.171 | | | 56.359 | | |
| **N** | 38 | | 34 | | | 72 | | |

*Averaged values from the 0.1, 0.3, 0.5, and 1.0 mg/kg/infusion sessions for mice in the cocaine paradigm and from the 0.01, 0.03, 0.1, 0.3, and 1.0 mg/kg/infusion sessions for mice in the remifentanil paradigm.

^1^Based on initial eigenvalues.

**Table S9. Component loadings of variables in novelty-induced open field measures from principal component analysis – Supporting Figure 6.**

| **Variable** |  | | | | |
| --- | --- | --- | --- | --- | --- |
|  | **Factor 1** | **Factor 2** | **Factor 3** | **Factor 4** | **Factor 5** |
| Distance | -0.474 | -0.268 | 0.018 | -0.414 | 0.473 |
| Vertical Episodes | -0.421 | -0.351 | 0.131 | 0.822 | 0.004 |
| Center Time | -0.222 | 0.613 | 0.737 | 0.033 | 0.171 |
| Rest Time | 0.473 | -0.234 | 0.137 | 0.142 | 0.780 |
| Stereotypic Episodes | -0.302 | 0.543 | -0.645 | 0.192 | 0.373 |
| Velocity | -0.484 | -0.280 | 0.067 | -0.307 | -0.015 |
| **Eigenvalues^1^** | 3.74 | 1.01 | 0.83 | 0.31 | 0.16 |
| **% Variance^1^** | 61.44 | 16.50 | 13.54 | 5.16 | 2.66 |
| **Cumulative %^1^** | 99.3 | | | | |
| **N** | 65 | | | | |

^1^Based on Initial eigenvalues.

**Table S10. Component loadings of variables in drug taking principal component analyses – Supporting Figure 6.**

| **Variable** | **Within-Drug Cocaine** | | | | | **Within-Drug Remifentanil** | | | | |
| --- | --- | --- | --- | --- | --- | --- | --- | --- | --- | --- |
|  | **Factor 1** | **Factor 2** | **Factor 3** | **Factor 4** | **Factor 5** | **Factor 1** | **Factor 2** | **Factor 3** | **Factor 4** | **Factor 5** |
| Acquisition sessions required | 0.144 | 0.420 | 0.400 | -0.593 | 0.528 | -0.114 | -0.374 | 0.122 | -0.736 | 0.534 |
| Reinforcements | -0.471 | 0.157 | 0.243 | -0.302 | -0.448 | -0.519 | -0.082 | 0.152 | -0.196 | -0.424 |
| Active lever responding | -0.504 | 0.136 | 0.254 | 0.049 | -0.231 | -0.514 | -0.056 | 0.343 | -0.013 | -0.247 |
| Inactive lever responding | -0.063 | 0.585 | -0.365 | -0.115 | -0.260 | -0.231 | 0.609 | 0.234 | -0.164 | 0.020 |
| Percent time-out responses | -0.245 | 0.414 | 0.259 | 0.708 | 0.368 | -0.135 | -0.116 | 0.600 | 0.542 | 0.508 |
| Lever accuracy | -0.194 | -0.473 | 0.49 | -0.052 | 0.033 | -0.051 | -0.679 | -0.046 | 0.201 | -0.276 |
| Latency to first lever response | 0.428 | 0.108 | 0.444 | 0.130 | -0.307 | 0.429 | -0.067 | 0.489 | -0.141 | -0.213 |
| Latency to first reinforcement | 0.467 | 0.176 | 0.279 | 0.146 | -0.410 | 0.442 | 0.012 | 0.435 | -0.195 | -0.307 |
| **Eigenvalues^1^** | 3.28 | 2.17 | 1.59 | 0.59 | 0.43 | 3.08 | 1.98 | 1.41 | 0.96 | 0.60 |
| **% Variance^1^** | 39.66 | 26.22 | 19.23 | 7.15 | 5.14 | 37.41 | 24.00 | 17.15 | 11.65 | 7.31 |
| **Cumulative %^1^** | 97.4 | | | | | 97.5 | | | | |
| **N** | 31 | | | | | 34 | | | | |

^1^Based on Initial eigenvalues.

**Table S11. Linear regression models for drug seeking – Supporting Figure 6.**

|  |  | **Within-Drug Cocaine** | | | **Within-Drug Remifentanil** | | |
| --- | --- | --- | --- | --- | --- | --- | --- |
|  |  | **Early Extinction** | **Late Extinction** | **Reinstatement** | **Early Extinction** | **Late Extinction** | **Reinstatement** |
| Linear Regression Coefficients | Intercept | 543.1796*** | 488.63*** | 50.689*** | 490.77*** | 774.511*** | 65.064*** |
|  | Drug-taking PC 1 | -75.1991*** | N/I | N/I | -52.70** | N/I | N/I |
|  | Drug-taking PC 2 | 29.2839 | N/I | N/I | N/I | 139.643** | 8.610* |
|  | Drug-taking PC 3 | 21.0105 | N/I | 2.394 | 79.06** | N/I | N/I |
|  | Drug-taking PC 4 | -66.7308* | N/I | N/I | N/I | N/I | N/I |
|  | Drug-taking PC 5 | N/I | N/I | -29.606** | N/I | N/I | N/I |
|  | Open field PC 1 | N/I | N/I | N/I | N/I | N/I | N/I |
|  | Open field PC 2 | N/I | N/I | N/I | 100.53* | N/I | N/I |
|  | Open field PC 3 | -120.2241** | 113.89 | N/I | N/I | N/I | N/I |
|  | Open field PC 4 | 54.6585 | N/I | N/I | 70.34 | N/I | N/I |
|  | Open field PC 5 | 162.0896** | N/I | N/I | 199.17** | 392.030* | 43.27** |
|  | Cocaine-induced hyperlocomotion^¥^ | -0.7613* | N/I | N/I | N/A | N/A | N/A |
|  | Remifentanil-induced hyperlocomotion^¥^ | N/A | N/A | N/A | N/I | -8.848. | N/I |
| Performance | **Residual standard error** | 91.19 | 218.8 | 24.43 | 136.1 | 349.8 | 28.06 |
|  | **Multiple R-squared** | 0.7697 | 0.1073 | 0.369 | 0.6829 | 0.4321 | 0.4293 |
|  | **Adjusted R-squared** | 0.6469 | 0.06677 | 0.309 | 0.6074 | 0.358 | 0.3817 |
|  | **F statistic** | 6.266 | 2.646 | 6.142 | 9.044 | 5.833 | 9.025 |
|  | **Degrees of Freedom** | 15 | 22 | 21 | 21 | 23 | 24 |
|  | **P value** | 0.00118 | 0.118 | 0.00794 | 0.000105 | 0.00407 | 0.00120 |

Significance codes: *p<0.05, **p<0.01, ***p<0.001.

Abbreviations: N/I, not included in the model based on Akaike information criterion; N/A, not applicable.

^¥^Total distances value for 20 mg/kg (i.p.) cocaine and 1 mg/kg (i.p.) remifentanil.
